# Sleep duration, vital exhaustion and perceived stress among pregnant migraineurs and non-migraineurs

**DOI:** 10.1186/1471-2393-10-72

**Published:** 2010-11-03

**Authors:** Michelle A Williams, Sheena K Aurora, Ihunnaya O Frederick, Chunfang Qiu, Bizu Gelaye, Swee May Cripe

**Affiliations:** 1 Department of Epidemiology, School of Public Health, University of Washington, Seattle, Washington, USA; 2 Center for Perinatal Studies, Swedish Medical Center, Seattle Washington, USA; 3 Swedish Headache Center, Seattle Washington, USA

## Abstract

**Background:**

Migraine has been associated with sleep disorders in men and non-pregnant women, but little is known about sleep complaints among pregnant migraineurs.

**Methods:**

A cohort of 1,334 women was interviewed during early pregnancy. At the time of interview we ascertained participants' migraine diagnosis status and collected information about sleep duration before and during early pregnancy, daytime sleepiness, vital exhaustion and perceived stress during early pregnancy. Multivariable logistic regression procedures were used to estimate odds ratios (ORs) and 95% confidence intervals (CIs) of short/long sleep duration, excessive daytime sleepiness, vital exhaustion and elevated perceived stress associated with a history of migraine.

**Results:**

Approximately 19.4% of the cohort (n = 259) reported having a medical diagnosis of migraine prior to the study pregnancy. Compared with women without migraine, the multivariable-adjusted ORs (95% CI) among migraineurs for short sleep duration before and during early pregnancy were 1.51 (1.09-2.09), and 1.57 (1.11-2.23), respectively. The corresponding OR (95% CI) for long sleep duration before and during pregnancy were 1.33 (0.77-2.31) and 1.31 (0.94-1.83), respectively. A modest and statistically insignificant association between migraine history and excessive daytime sleepiness in early pregnancy was noted (OR = 1.46; 95% CI 0.94-2.26). Migraineurs had an increased risk of vital exhaustion (OR = 2.04; 95% CI 1.52-2.76) and elevated perceived stress (OR = 1.57; 95% CI 1.06-2.31). Observed associations were more pronounced among overweight migraineurs.

**Conclusions:**

These data support earlier research documenting increased risks of sleep disorders among migraineurs; and extends the literature to include pregnant women. Prospective studies are needed to more thoroughly explore factors that mediate the apparent migraine-sleep comorbidity among pregnant women.

## Background

Sleep disturbance, a common complaint among patients with migraine, has been identified as a precipitating factor for headache attacks in some studies [1,2]. A substantial literature indicates that sleep disturbance and migraine are closely related, with the latter having considerable impacts on sleep quality [1-4]. Notably, sleep has also been reported to be important in relieving migraine in some patients [1].

Migraine prevalence is greatest in women of reproductive years [5-7]; and although many pregnant migraineurs show improvement or resolution of migraine during pregnancy, migraine continues to be a significant problem for over 33% of pregnant migraineurs [5]. The prevalence of migraine is known to be high among reproductive age women and pregnant women [5-7]; and pregnancy associated physiological and hormonal changes are known to contribute to increased prevalence and severity of sleep disorders and complaints among pregnant women [8,9]. However, despite these observations, little is known about sleep disorders among pregnant women with migraine. Furthermore, to the best of our knowledge, no studies have investigated associations of migraine with sleep duration and quality among pregnant women. Despite awareness of pregnancy associated metabolic and morphological changes that contribute to poor and fragmented sleep during pregnancy, little has been done to properly and comprehensively assess the influence of disturbed sleep on perinatal and maternal health. To fill this gap in the literature, we assessed the relative risks of short and long sleep duration (before and during pregnancy), as well as excessive daytime sleepiness, vital exhaustion and elevated perceived stress during pregnancy among women with and without a history of migraine. We hypothesized that pregnant women with a history of migraine were more likely than women with no history of migraine to have shorter sleep durations, and to have higher risks of excessive daytime sleepiness, vital exhaustion and perceived stress.

## Methods

### Study population and setting

This analysis is based on data collected from a cohort of women attending prenatal care clinics (for routine prenatal care) affiliated with Swedish Medical Center in Seattle, Washington, USA. Eligible women started prenatal care before 20 weeks gestation, were 18 years of age or older, could speak and read English, and planned to carry the pregnancy to term and to deliver at the hospital. At between 8-19 weeks (mean and standard deviation: 16 ± 2.6) weeks gestation, participants reported sociodemographic, behavioral, and health characteristics in a structured interview. After delivery, study personnel abstracted data from participants' hospital labor and delivery medical records and clinic records. Between December 2003 and July 2006, 1,393 (82%) of 1,685 approached women consented to participate. We sequentially excluded 12 women with early pregnancy losses prior to the interview and 47 women who did not complete the interview. Thus, 1,334 women remained for analysis. All study procedures were approved by the Institutional Review Board of Swedish Medical Center. All participants provided written informed consent.

### Data Collection

Interviewer-administered questionnaires were completed by participants in the analytical population at a mean gestational age of 16 weeks. Characteristics assessed using the questionnaire (i.e., self-administered) included maternal age, height, pre-pregnancy weight, reproductive and medical history including her history of migraine, average nightly sleep duration (before and during pregnancy), vital exhaustion during early pregnancy, and perceived stress during early pregnancy. Maternal history of *migraine diagnosis *was determined by response to the questions "Has a doctor ever told you that you have migraine headache?" and "If so, how old were you when your doctor gave you this diagnosis?" Maternal *average nightly sleep duration *before and during pregnancy was ascertained by asking women the following questions: (1) "During the year prior to this pregnancy, how many hours per night did you sleep?" and (2) "Since becoming pregnant, how many hours per night do you sleep?" Responses were reported as integers. For bivariate analyses, we classified participants into 4 sleep duration categories: ≤ 6, 7, 8, and ≥ 9 hours, respectively. For multivariate analyses, we classified participants as short (≤ 6 hours); normal (7-8 hours); and long (≥ 9 hours) duration sleepers. These categorizations were decided upon *a priori*, as decisions were guided by cut-points used by previously investigators, particularly those who focused on sleep problems among pregnant migraineurs [1].

Maternal report of *vital exhaustion *in early pregnancy was ascertained by asking women:

"Since becoming pregnant, how often did you experience a sense of exhaustion (except after exercise)?" Response choices were: (1) never; (2) somewhat infrequently (about once monthly); (3) frequent (2-3 times per month); and (4) very frequent (almost weekly). For multivariable analyses, we collapsed responses into a dichotomous variable with "no" comprising the responses never and somewhat infrequently, and "yes" comprising the responses frequent and very frequent.

We used the Epsworth Sleepiness Scale (ESS) [10] to assess maternal daytime sleepiness status during early pregnancy. Participants were asked to rate, on a scale of 0 to 3 (where 0 = would never doze; 1 = slight chance of dozing; 2 = moderate chance of dozing; and 3 = high chance of dozing), the likelihood they would fall asleep or doze off in each of the following eight common situations: sitting and reading; watching television; sitting, inactive in a public place (e.g., a theater or meeting); as a passenger in a car for an hour without a break; lying down to rest in the afternoon when circumstances permit; sitting and talking to someone; sitting quietly after lunch without alcohol; and in a car, while stopped for a few minutes in traffic. The scale yields a total score that ranges from 0 to 24, with higher ESS scores representing more severe subjective daytime sleepiness, respectively. Scores of 0-9, 10-12 and 13-24 are considered to represent normal, borderline and abnormal daytime sleepiness. For multivariable analyses, we created a dichotomous variable where participants with scores of ≥13 were classified as having excessive daytime sleepiness. We used a modified abbreviated version of the *Perceived Stress Scale (PSS)*, to measure the subjective experiences of stress and coping with stress using the past three month as timeframe. This timeframe corresponds to the time period since becoming pregnant (i.e., the first trimester). The abbreviated version, an economical and simple psychological instrument to administer, measures the degree to which situations in participants' life over the period of observation are appraised as stressful [11]. Items were selected to detect how unpredictable, uncontrollable, and overloaded participants find their lives. For multivariate analyses, we classified participants with scores ≥ 7 as having elevated perceived stress; those participants with scores <7 served as the reference group.

#### Statistical analytical methods

We compared the frequency distribution of sociodemographic, lifestyle, behavioral and medical history characteristics of participants according to whether or not they had received a physician diagnosis of migraine prior to the index pregnancy. We used unadjusted and multivariable-adjusted logistic regression models to calculate odds ratios (ORs) and 95% confidence intervals (CIs) of the association between migraine and sleep or stress variables. Separate models were fitted for each sleep complaint or stress. In multivariable models, we adjusted for maternal age (continuous), parity (nulliparous, multiparous), history of pre-gestational hypertension (no, yes), and pre-pregnancy body mass index (≤ 18.5, 18.5-24.9, 25-29.9, ≥ 30 kg/m^2^). Additional adjustment for the other covariates listed in Table 1 (including maternal age) did not substantially change the effect estimates. We evaluated the joint effect of migraine history and pre-pregnancy overweight status. We classified women by the joint distribution of prior history of migraine diagnosis (no vs. yes) and pre-pregnancy overweight status (< 25 vs. ≥ 25 kg/m^2^) resulting in the following categories: no migraine and lean; history of migraine and lean; no migraine and overweight; and history of migraine and overweight. This analytical approach allowed estimating ORs for sleep complaints among lean women with migraine (i.e., to isolate the effect of migraine, independent of overweight/obese status) and the ORs for sleep complaints among overweight women without migraine (i.e. to isolate the effect of overweight status, independent of migraine status) when using lean women without migraine as the referent group. The joint effect (or combined effect of both migraine and overweight status) is determined by comparing those positive for both characteristics with the referent group.

**Table 1:** Characteristics of the study population according to migraine status, Seattle, Washington, USA, 2003-2006

	Physician Diagnosed Migraine		
		
	Yes	No	
	N = 259	N = 1,075	p-value
**Characteristics**	**%**	**%**	

Maternal Age (years)	33.2 ± 4.5	33.4 ± 4.4	0.47
< 35	61.8	59.6	0.53
≥ 35	38.2	40.4	
Non-Hispanic white race/ethnicity	89.6	87.4	0.26
Annual household income (US$)			
< 30,000	2.7	1.8	0.61
30,000-69,999	12.0	14.3	
≥ 70,000	80.7	79.4	
Missing	4.6	4.6	
Nulliparous	53.7	60.9	0.03
Unmarried	11.2	7.6	0.06
Pre-gestational chronic hypertensive	8.1	3.1	< 0.001
Family history of diabetes mellitus	13.9	14.6	0.77
Family history of hypertension	54.4	49.3	0.14
Employed during pregnancy	81.9	77.9	0.16
Smoked during pregnancy	4.6	5.2	0.71
No prenatal vitamin	3.1	2.4	0.54
No exercise during pregnancy	7.3	7.4	0.99
Pre-pregnancy body mass index (kg/m^2^)*	24.4 ± 5.7	23.3 ± 4.3	< 0.001
Normal (18.5-24.9)	63.3	71.5	0.02
Lean (< 18.5)	4.3	4.7	
Overweight (25-29.9)	20.8	17.2	
Obese (≥ 30)	11.6	6.6	

Mean ± standard deviation (SD).

*P-value < 0.05 from Student t test for continuous variable or from Chi-square test for categorical variables.

All analyses were performed using Stata 9.0 statistical software (Stata, College Station, TX). All continuous variables are presented as mean ± standard deviation (SD). All reported confidence intervals were calculated at the 95% level. All reported p-values are two-tailed.

## Results

Women with a prior history of physician diagnosed migraine were more likely to be multiparous, to have a history of pre-gestation chronic hypertension and to be overweight and obese when compared with women who did not have a history of migraine (Table 1). Other characteristics including marital status, annual household income, race/ethnicity, physical activity and multivitamin use during pregnancy were similar for women with and without a history of migraine. Descriptive statistics of maternal sleep complaint and stress variables are summarized in Table 2. Women with a history of migraine were more likely to report short sleep duration (≤ 6 hours) before and during pregnancy, as compared with those with no migraine history. Similarly migraineurs reported more frequent feeling of vital exhaustion and their perceived stress scores were higher than those reported by non-migraineurs. The two groups were similar with respect to their Epsworth sleepiness scores.

**Table 2:** Sleep complaints reported by pregnant women with and without a medical history of migraine, Seattle, Washington, USA, 2003-2006

	Physician Diagnosed Migraine		
		
	Yes	No	
	N = 259	N = 1,075	
**Sleep Complaints**	**Mean ± SD or %**	**Mean ± SD or %**	**P-value**

Sleep hours before pregnancy (hours)	7.2 ± 1.0	7.4 ± 0.9	0.01*
≤ 6	27.0	18.5	0.03**
7	35.5	38.4	
8	29.4	35.4	
≥ 9	6.9	6.0	
Missing	1.2	1.7	
			
Sleep hours during pregnancy (hours)	7.7 ± 1.4	7.8 ± 1.3	0.30*
≤ 6	24.7	17.2	0.01**
7	19.7	23.2	
8	28.2	34.4	
≥ 9	26.2	24.8	
Missing	1.2	0.4	
			
Epworth sleepiness scale during pregnancy (score)	7.9 ± 3.5	7.5 ± 3.4	0.20*
Normal (0-9)	68.7	72.7	0.20**
Borderline (10-12)	18.9	18.9	
Abnormal (13-24)	12.4	8.3	
Missing	0.0	0.1	
			
Vital exhaustion during pregnancy			
Never	39.0	54.4	< 0.001**
Infrequently	24.3	24.0	
Frequently	16.2	8.8	
Very frequently	19.7	12.0	
Missing	0.8	0.8	
			
Perceived stress scale (score)	4.2 ± 2.4	3.7 ± 2.3	0.008*
0-3	41.7	47.4	0.07**
4-6	39.8	39.6	
7-12	16.2	10.6	
Missing	2.3	2.4	

*P-value from Student's t test for continuous variables

**P-value from Chi-square test for categorical variables

**Figure 1:**
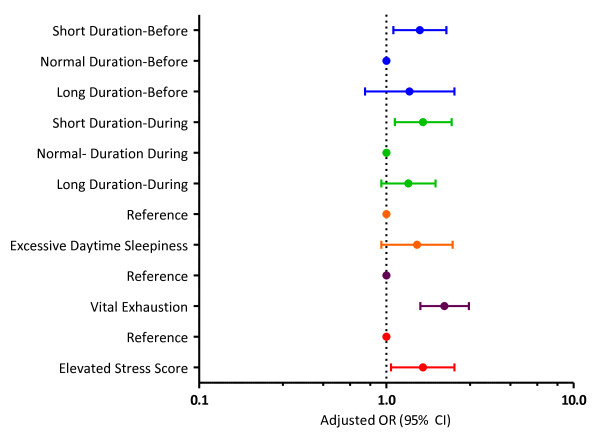
**Adjusted odds ratios (AOR) and 95% confidence intervals (CI) of risk of short/long sleep duration, excessive daytime sleepiness, vital exhaustion and elevated perceived stress according to maternal history of migraine**. Odds ratios are adjusted for parity, history of pre-gestational hypertension and pre-pregnancy body mass index.

Women with a history of migraine were more likely to report sleeping ≤ 6 hours nightly before pregnancy (OR = 1.66; 95% CI 1.21-2.29) than those without the history. After adjusting for maternal parity, history of pre-gestational hypertension and pre-pregnancy body mass index, the association was attenuated but remained statistically significant (AOR = 1.51; 95% CI 1.09-2.09). Migraineurs were more likely to report short (≤ 6 hours, AOR = 1.57; 95% CI 1.11-2.23) and long (≥ 9 hours, AOR = 1.31; 95% CI 0.94-1.83) sleep duration during pregnancy than their non-migraine counterparts. The adjusted odds of excessive daytime sleepiness (AOR = 1.46; 95% CI 0.94-2.26), vital exhaustion (AOR = 2.04; 95% CI 1.52-2.76) and elevated perceived stress during pregnancy (AOR = 1.57; 95% CI 1.06-2.31) were elevated among migraineurs compared with non-migraineurs, though only the latter two conditions reached statistical significance (Table 3 and Figure 1)

**Table 3:** Odds ratios (OR) and 95% confidence intervals (CI) of short/long sleep duration, excessive daytime sleepiness, vital exhaustion and elevated perceived stress according to maternal history of migraine, Seattle, Washington, USA, 2003-2006

	Migraine Diagnosis			
			
	Yes	No		
	N = 259	N = 1,075	Unadjusted	Adjusted*
			
Sleep Complaint	%	%	OR (95% CI)	OR (95% CI)
Sleep duration before pregnancy				
Short (≤ 6 hours)	27.0	18.5	1.66 (1.21-2.29)	1.51 (1.09-2.09)
Normal (7-8 hours)	64.9	73.8	1.00 (Reference)	1.00 (Reference)
Long (≥ 9 hours)	6.9	6.0	1.33 (0.77-2.30)	1.33 (0.77-2.31)
				
Sleep duration during pregnancy				
Short (≤ 6 hours)	24.7	17.2	1.73 (1.22-2.43)	1.57 (1.11-2.23)
Normal (7-8 hours)	47.9	57.6	1.00 (Reference)	1.00 (Reference)
Long (≥ 9 hours)	26.2	24.8	1.27 (0.92-1.77)	1.31 (0.94-1.83)
				
Excessive daytime sleepiness during pregnancy				
No	87.6	91.7	1.00 (Reference)	1.00 (Reference)
Yes	12.4	8.3	1.56 (1.02-2.40)	1.46 (0.94-2.26)
				
Vital exhaustion during pregnancy				
No	63.3	78.4	1.00 (Reference)	1.00 (Reference)
Yes	35.9	20.8	2.14 (1.60-2.88)	2.04 (1.52-2.76)
				
Elevated perceived stress score (≥7) during pregnancy				
No	81.5	87.0	1.00 (Reference)	1.00 (Reference)
Yes	16.2	10.6	1.63 (1.11-2.40)	1.57 (1.06-2.31)

*Adjusted for parity, history of pre-gestational hypertension and pre-pregnancy body mass index.

**Column percentages may not add up to 100% due to missing values.

We evaluated the joint effect of migraine history and pre-pregnancy overweight status (Table 4) and noted that overweight migraineurs had the highest odds of short sleep duration before and during pregnancy, excessive daytime sleepiness, vital exhaustion and elevated perceived stress during pregnancy. Compared with lean women without migraine, the multivariable-adjusted ORs among overweight migraineurs for short sleep duration before and during early pregnancy were 2.96 (95% CI 1.77-4.95), and 2.43 (95% CI 1.40-4.21), respectively. Statistically significant associations were also observed for long sleep duration before pregnancy (AOR = 2.37; 95% CI 1.05-5.36), excessive daytime sleepiness (AOR = 2.31; 95% CI 1.21-4.42), vital exhaustion (AOR = 2.80; 95% CI 1.73-4.55) and elevated perceived stress (AOR = 2.55; 95% CI 1.42-4.59) during pregnancy.

**Table 4:** Adjusted odds ratios (AOR) and 95% confidence intervals (CI) of short/long sleep duration, excessive daytime sleepiness, vital exhaustion and elevated perceived stress according to maternal history of migraine and pre-pregnancy overweight status**, Seattle, Washington, USA, 2003-2006

	No Migraine		Yes Migraine		No Migraine		Yes Migraine	
	No Overweight		No Overweight		Yes Overweight		Yes Overweight	
	(N = 819)		(N = 175)		(N = 256)		(N = 84)	
**Sleep Complaint**	**%**	**AOR (95% CI)**	**%**	**AOR (95% CI)**	**%**	**AOR (95% CI)**	**%**	**AOR (95% CI)**

Sleep duration before pregnancy								
Short (≤ 6 hours)	16.9	1.00 (Reference)	22.9	1.38 (0.92-2.08)	23.8	1.58 (1.11-2.24)	35.7	2.96 (1.77-4.95)
Long (≥ 9 hours)	6.1	1.00 (Reference)	5.7	1.01 (0.50-2.05)	5.5	1.03 (0.55-1.91)	9.5	2.37 (1.05-5.36)
								
Sleep duration during pregnancy								
Short (≤ 6 hours)	16.0	1.00 (Reference)	21.1	1.51 (0.98-2.33)	21.1	1.43 (0.98-2.08)	32.1	2.43 (1.40-4.21)
Long (≥ 9 hours)	24.5	1.00 (Reference)	28.6	1.45 (0.98-2.13)	25.8	1.19 (0.85-1.67)	21.4	1.19 (0.66-2.16)
								
Excessive daytime sleepiness during pregnancy	7.6	1.00 (Reference)	10.3	1.40 (0.80-2.43)	10.5	1.41 (0.87-2.28)	16.7	2.31 (1.21-4.42)
								
Vital exhaustion during pregnancy	19.3	1.00 (Reference)	33.7	2.08 (1.45-2.98)	25.4	1.44 (1.03-2.02)	40.5	2.80 (1.73-4.55)
								
Elevated perceived stress during pregnancy	9.8	1.00 (Reference)	13.7	1.44 (0.88-2.35)	13.3	1.44 (0.94-2.23)	21.4	2.55 (1.42-4.59)

*Adjusted for parity, and history of pre-gestational hypertension.

**None of the interactions reached statistical significance.

## Discussion

Approximately 19.4% of the cohort reported having a medical diagnosis of migraine prior to the study pregnancy. Overall, migraineurs were more likely than non-migraineurs to report short sleep durations, vital exhaustion and elevated perceived stress. The odds of these complaints were particularly elevated among overweight migraineurs.

As recently, reviewed by Rains and Poceta [3], no epidemiological studies to date have examined the comorbidity of headache (by specific International Headache Society diagnoses [12] and the complete spectrum of sleep disorders in the general population. Nevertheless, several studies, generally conducted in men and non-pregnant women [1,13-17] or children [18,19] have examined one or more aspects of the headache-sleep comorbidity spectrum. Our results are in accordance with previous reports indicating an increased prevalence of sleep disorders, stress or fatigue among individuals with headaches or migraine; and extend this literature to include observations of such associations in pregnant women. For instance, our observation of positive associations of short sleep duration with migraine is consistent with reports from Kelman and Raines [1] who reported that over a third of migraineurs reported difficulty initiating and maintaining a healthy sleep pattern. Notably, 38% of participants reported sleeping an average of ≤ 6 hours per night, and were more likely to suffer more frequent and more severe headaches. Habitual short sleep duration (5.6 hours), consistent with insomnia, was also observed among headache patients enrolled in small clinical studies [14,20]. Rothrock et al [20] reported that short sleep duration was more common among chronic (43.2%) versus episodic (18.9%) headache patients. Boardman et al [17], in their cross-sectional postal survey of 2,662 British adults from the general population, reported that headache (of all types) occurrence was associated with reported sleep problems, stress and fatigue. After adjusting for participants' age and gender, the authors observed a positive trend of headache occurrence with increasing severity of sleep problems. Subjects reporting headaches in the 3-month period prior to assessment were more than twice as likely to report slight sleep problems (AOR = 2.4; 95% CI 1.7-3.2) as compared with controls who did not suffer headaches. The corresponding ORs and 95% confidence intervals were (AOR = 3.6; 95% CI 2.6-5.0) for moderate and (AOR = 7.5; 95% CI 4.2-13.4) severe sleep problems. Recently, Vgontzas et al [21] noted that adults with migraine reported having significantly more lifetime sleeping problems (OR = 2.35; 95% CI 1.1-4.6), more current complaints of inadequate sleep (OR = 2.5; 95% CI 1.2-5.0), and difficulty falling asleep (OR = 3.0; 95% 1.5-6.3) than those without migraine. Overall, our observations of higher odds of reported short sleep duration, excessive daytime fatigue, vital exhaustion and stress are consistent with previous studies. Moreover, our findings and those of others [17,21] are biologically plausible. We provide a brief summary of clinical studies that support observed statistical associations.

Investigators [2,22] speculate that common underlying pathophysiological neuroendocrine alterations involving the hypothalamus, serotonin and melatonin synthesis and secretion may, in part, explain consistent observations of increased risks of sleep disorders in patients with migraine and other primary headache disorders. This hypothesis is supported by results from clinical [23-25] and functional neuroendocrine imaging studies [26]. Investigators have documented altered melatonin concentrations in patients with migraine [23] and chronic headaches [25]. Moreover, investigators have reported lower melatonin concentrations in patients with comorbid migraine and insomnia [27]. Melatonin, known to play a role in the biological regulation of circadian rhythms and sleep, has been shown to be effective in preventing migraine and chronic headaches in adults and children [24,27]. Alterations in the serotonergic system have also been implicated in the pathogenesis of comorbid migraine and sleep disorders [2,28]. Serotonin secreted from the dorsal raphe nuclei is implicated in both the control of sleep cycles and migraine pathogenesis [29,30]. The suprachiasmatic nucleus of the hypothalamus regulates the release of serotonin, supporting the thesis that the hypothalamus likely plays an important role in both the control of nociception and sleep-regulating systems.

Several limitations of our study merit discussion and consideration. First, maternal migraine status was based on self-reports made during interviews and on medical records review. Although the American Migraine Study II [31] and the Women Health Study [32] document good agreement between migraine classification based on self reports with International Headache Society classification criteria, we cannot exclude the possibility of that migraine status was underreported in our study. Studies that systematically use screening and confirmatory diagnostic evaluations will attenuate greatly concerns about misclassification of maternal migraine diagnoses in epidemiological studies. We were also unable to differentiate migraineurs on the basis of features such as migraine with aura, migraine frequency and timing of most recent attacks. Migraine with and without aura are likely to have distinct pathologies and implications [33] and should be disaggregated in future studies. Second, maternal habitual sleep duration before and during pregnancy was obtained from self-report, and thus is likely susceptible to misclassification. Reported sleep duration is known to be only moderately correlated with wrist actigraph-measured sleep duration (*r *= 0.47), and reports are generally longer by approximately 34 minutes for each hour of objectively measured sleep duration [34]. Future studies will require making objective assessments of maternal sleep duration. Third, we did not have information concerning participants' shift-work or insomnia status and thus cannot attribute observed associations of short sleep duration with migraine to occupational or medical conditions associated with short sleep duration. For instance, though related, short sleep duration and insomnia are different entities. Insomnia entails dissatisfaction with the quality of sleep and an inability to sleep given adequate opportunity. Insomnia can result in short sleep duration, but individuals with short sleep duration do not necessarily suffer from insomnia (i.e., participants may sleep less because they choose to do so or because they lack the opportunity to sleep). Future studies that allow for the comprehensive ascertainment of maternal sleep disorders (e.g., sleep disordered breathing, restless legs syndrome, insomnia, and circadian rhythm disorders) will be needed to more thoroughly assess the co-morbidity of migraine and sleep disorders among pregnant women. Fourth, although we adjusted for several potential confounders, we cannot exclude the possibility of residual confounding due to misclassification of adjusted variables (e.g., maternal pre-pregnancy body mass index) or confounding by other unmeasured variables (e.g., maternal psychiatric disorders or the severity and frequency of migraine episodes during pregnancy). In consideration of evidence suggesting that adiposity may be associated with both migraine and sleep disorders [21,35], we report results from multivariable models which allow for assessing the independent and joint effects of migraine and overweight status on each sleep complaint variable. Lastly, the generalizability of our study may also be limited as our cohort was primarily comprised of Non-Hispanic White and well-educated women. The concordance of our results with those from other studies that have included racially, ethnically and geographically diverse populations [1,13-19], however, serve to attenuate these concerns.

## Conclusions

In summary, we found increased risks of short sleep duration, excessive daytime sleepiness, vital exhaustion and perceived stress among pregnant women with migraine compared with pregnant women without migraine. These associations were particularly strong among overweight migraineurs. Despite noted study limitations, our results are consistent with a larger body of work documenting associations between migraine and sleep disturbances in men, non-pregnant women and children. Large well designed prospective cohort studies that allow for the comprehensive examination of comorbidity of the full spectrum of headache and migraine disorders (e.g., as specified by the International Headache Society) and the full spectrum of sleep disorders in pregnant women are warranted. Such studies should include objective assessments of sleep duration and disorders and should include comprehensive assessments of environmental, behavioral, and genetic risk factors of headache, migraine and sleep disorders. Enhanced understanding of the epidemiology and shared pathophysiological mechanisms between headaches and sleep disturbances are expected to provide important information needed for enhancing the diagnosis and treatment of these disorders in pregnant women.

## Competing interests

The authors declare that they have no competing interests.

## Authors' contributions

MAW had full access to all the data in the study and takes responsibility for the integrity of the data, the accuracy of the data analysis, and the decision to submit for publication. MAW conceived, designed and obtained funding for the study. CQ analyzed the data. MAW, SKA and CQ drafted the manuscript. All authors interpreted the data, critically revised the draft for important intellectual content, and gave final approval of the manuscript to be published.

## Pre-publication history

The pre-publication history for this paper can be accessed here:

http://www.biomedcentral.com/1471-2393/10/72/prepub
